# The relationship between the atherogenic index of plasma and hyperuricemia in American adults aged over 20 years: A cross-sectional study

**DOI:** 10.1371/journal.pone.0344977

**Published:** 2026-03-19

**Authors:** Chunjie She, Liuyang Shi, Yang Li, Linhan Qin, Nan Fang, Kehai Shi, Zhongping Miao, Hefeng Liu

**Affiliations:** Department of Orthopaedics, The Fourth Affiliated Hospital of Anhui Medical University, Chaohu, Anhui, China; Università degli Studi di Milano, ITALY

## Abstract

**Background:**

The presence of hyperuricemia (HUA) is closely associated with lipid disorders and the development of cardiovascular disease (CVD). However, research on the relationship between the atherogenic index of plasma (AIP) and HUA remains limited among the general adult population in the United States. This study aims to elucidate the association between the AIP and HUA using data from a nationally representative database in the United States.

**Methods:**

This study included a total of 7,057 participants, with data obtained from the National Health and Nutrition Examination Survey (NHANES) spanning 2011–2018. The AIP was calculated as log10 (triglycerides/high-density lipoprotein cholesterol). HUA served as the outcome variable, defined by serum uric acid (SUA) levels. Multivariate logistic regression, generalized additive models, smoothing fitting curves, subgroup analyses, and interaction tests were employed to reveal the relationship between AIP and HUA.

**Results:**

After adjusting for all covariates, a statistically significant positive correlation was observed between AIP and the odds of HUA (OR = 3.22, 95%CI [2.54, 4.10], P < 0.001). Participants in the highest AIP quartile (Q4) had a 1.76-fold higher risk of HUA compared to those in the reference AIP quartile (Q1) (OR = 2.76, 95%CI [2.20, 3.45], P < 0.001). Stratified analyses confirmed that the positive correlation between AIP and HUA risk was significant and consistent, regardless of gender and body mass index (BMI) category. Additionally, the study found a nonlinear inverted L-shaped association between AIP and the risk of HUA, with the inflection point at 0.34. Subgroup analysis revealed that gender had a significant interaction with the AIP. Females showed a stronger association than males.

**Conclusions:**

AIP and the risk of HUA demonstrated an inverted L-shaped positive association in the adult US population. The association was stronger in females than in males.

## Introduction

The uric acid (UA) is the final product of purine nucleotide degradation. Under normal physiological conditions, UA production, intake, and excretion remain stable, and it is an important hydrophilic strong antioxidant in the human body [1]. However, when the metabolic balance is disrupted and SUA levels exceed the prescribed range, HUA occurs [2,3]. HUA not only acts as the primary etiology of gout but also participates in the pathogenesis of CVD, chronic kidney disease, diabetes, and lipid abnormalities, imposing a substantial burden on public health [4–7]. Globally, the prevalence of HUA generally maintains an increasing trend, with certain regional and economic variations [8,9]. Australasia and high-income North America are the most commonly affected regions [10]. According to NHANES data from 2015–2016, approximately 20% of the population in the United States experiences HUA, which is much higher than China’s rate of 11.1% during the same period [11,12]. The AIP proposed by Dobiásová et al in 2001 is a novel lipid marker that reflects the development of arteriosclerosis resulting from alterations in plasma lipoproteins [13]. As per the formula’s definition, it represents the triglycerides (TG) to high-density lipoprotein cholesterol (HDL-C) ratio; meanwhile, owing to its strong inverse correlation with low-density lipoprotein cholesterol (LDL-C) particle size, it can serve as an indirect metric of LDL-C particle diameter [13,14]. Compared to conventional lipid markers such as total cholesterol (TC), TG, HDL-C, and LDL-C, the AIP has demonstrated superior efficacy in assessing the risk of CVD and dyslipidemia [15]. It has been established as a robust biomarker for lipid metabolism and CVD events [14]. Furthermore, its link with Insulin resistance, non-alcoholic liver disease, and even bone density has been found in recent years [16–18]. Epidemiological studies have proven that both HUA and dyslipidemia are risk factors for CVD [19,20]. Considering the high prevalence of HUA and the elevated mortality rate of CVD in the United States, it is imperative to investigate the association between AIP and HUA [11,21]. We conducted a comprehensive literature search on PubMed, covering studies published up to September 8, 2024. The majority of research on AIP and HUA primarily focuses on the Chinese population. Only one single study conducted in a US population has reported on the association between the AIP and hypertension with HUA. This study revealed a positive correlation between AIP and HUA, which persisted within the hypertensive population, and additionally demonstrated that AIP exhibits good diagnostic efficacy for isolated HUA. However, since the study aimed to compare the association of hypertension plus HUA with seven anthropometric indices, and AIP demonstrated the poorest discriminative ability for hypertension with HUA, the relationship between AIP and HUA was overlooked and not elaborated in detail [22]. To address the limitations of previous research, this study has undertaken the following innovative work. Firstly, we revealed an inverted L-shaped relationship between AIP and HUA and calculated the inflection point. Secondly, we elucidated the correlation between AIP and HUA across diverse subgroups and performed interaction tests. Thirdly, we provided detailed stratified analyses of the association between AIP and HUA in key subgroups. We utilized data from the nationally representative dataset, the NHANES, to elucidate the correlation between AIP and HUA in the American adult population. It is worth noting that this study is a cross-sectional study, and therefore cannot establish a causal relationship.

## Methods

### Data source

The data utilized in this study were obtained from the publicly accessible National Health and Nutrition Examination Survey (NHANES), which is a program of studies designed to assess the health and nutritional status of the United States population. Annually, it conducts interviews, physical examinations, and laboratory tests on a nationally representative sample of approximately 5,000 individuals, with detailed and comprehensive records of clinical data and demographic characteristics. All collected data were anonymized and encoded before being released to the public.

### Ethics statement

The NHANES has received approval from the ethics review board of the National Center for Health Statistics and obtained written informed consent from all participants enrolled in the research.

### Study population

**Fig 1:**
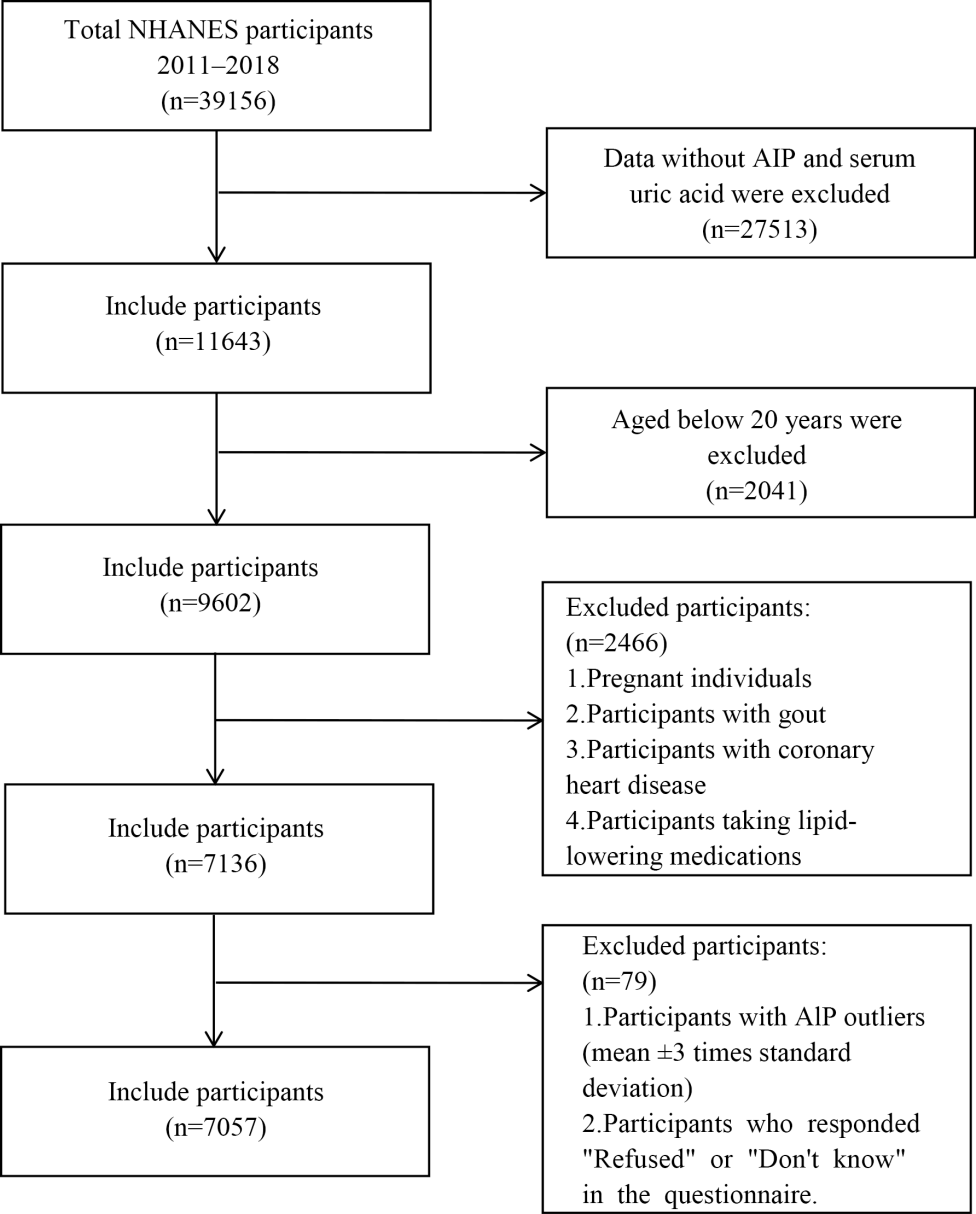
Flow chart of study population selection.

This study incorporated data from four NHANES cycles spanning the years 2011–2018, encompassing a total of 39,156 participants. Participants were required to be at least 20 years of age and to have complete data for all exposure and outcome variables (including TG, HDL-C, and SUA). Additionally, the following participants were excluded from the study: [1] Pregnant individuals. [2] Patients with gout. [3] Patients with coronary heart disease. [4] Patients taking lipid-lowering medications. After removing a few extreme values and undefined data, a total of 7057 patients were finally included in this study. The study population screening process is depicted in Fig 1.

### Definitions of the AIP and hyperuricemia

The AIP was defined as the logarithm of the ratio between TG and HDL-C, with a base 10 logarithm. The formula is expressed as lg (TG/HDL-C), where both TG and HDL-C are measured in mmol/L. The TG values in mg/dL were converted to mmol/L by multiplying by 0.01129. HDL-C in mg/dL was converted to mmol/L by multiplying by 0.02586. The measured AIP was divided into four groups by quartile: Q1 group (＜−0.34), Q2 group (−0.34,-0.12), Q3 group (−0.12,0.10), and Q4 group (＞0.10). HUA was defined by the level of SUA. When the SUA concentration exceeds 7.0 mg/dL in men and 5.7 mg/dL in women, HUA can be diagnosed [11].

### Acquisition of covariates

Based on previous research and clinical experience, we selected covariates potentially associated with AIP and HUA from the NHANES database, covering the following aspects: [1] Demographic characteristics: gender, race, age, height, weight, education level, waist circumference, the ratio of family income to poverty (PIR), and BMI. [2] Routine laboratory test results: Aspartate Aminotransferase, Alanine Aminotransferase, TC, LDL-C, Estimated Glomerular Filtration Rate (eGFR), serum creatinine, and blood urea nitrogen (BUN). [3] Lifestyle habits and personal medical history: smoking status, drinking status, physical activity, hypertension, and diabetes.

In the aforementioned covariates, age, gender, race, PIR, education, smoking status, drinking status, and physical activity were obtained through a self-report questionnaire. In the NHANES, participants’ ethnicity is primarily categorized into the following five major groups: Mexican American, Other Hispanic, Non-Hispanic white, Non-Hispanic black, and Other Race – Including Multi-Racial. Education was defined by the survey question: “What is the highest grade or level of school you have completed or the highest degree you have received?” and categorized into five levels: Less than 9th grade, 9–11th grade, High school graduate/GED or equivalent, Some college or AA degree, and College graduate or above. PIR was calculated by dividing family (or individual) income by the poverty guidelines specific to the survey year, which vary by family size and geographic location. According to the following two questions to define smoking status: “Have you smoked at least 100 cigarettes in your lifetime?” and “Do you smoke now?” The classification was as follows: no smoking (lifetime consumption < 100 cigarettes), quit smoking (lifetime consumption > 100 cigarettes but currently denying smoking), and current smoking (lifetime consumption > 100 cigarettes and admitting current smoking). The National Institute of Alcohol Abuse and Alcoholism (NIAAA) defined heavy drinking as more than four drinks for women and five drinks for men on a given day. Drinking status was categorized into low drinking and heavy drinking through the question: “Was there ever a time or times in your life when you drank 4/5 or more drinks of any kind of alcoholic beverage almost every day?”. According to the standards of the Physical Activity Guidelines for Americans, respondents are classified into two categories: active (engaging in more than 150 minutes of moderate-intensity physical activity per week, or more than 75 minutes of vigorous-intensity physical activity, or an equivalent combination of both) and insufficiently Active (failing to meet the above criteria). The time spent on moderate-intensity physical activities and vigorous-intensity activities was obtained by asking the following questions, respectively: “How much time do you spend doing moderate-intensity activities at work/moderate-intensity sports, fitness or recreational activities?” and “How much time do you spend doing vigorous-intensity activities at work/vigorous-intensity sports, fitness or recreational activities?”

The remaining covariates, including waist circumference, BMI, aspartate aminotransferase, alanine aminotransferase, serum creatinine, BUN, TC, eGFR, hypertension, and diabetes, were assessed through laboratory or physical examination. Detailed descriptions of laboratory methodology and data processing are available on the NHANES website. Taking BUN as an example, the method to measure BUN utilizes a coupled enzyme reaction (urease, followed by glutamate dehydrogenase), with measurement of NADH (converting to NAD+) occurring at 340 nm. BUN in mg/dL was converted to mmol/L by multiplying by 0.357. BMI was calculated as weight in kilograms divided by height in meters squared. According to the World Health Organization (WHO) classification criteria, BMI was further categorized into three groups: normal weight (BMI < 25), overweight (25 ≤ BMI < 30), and obesity (BMI ≥ 30). eGFR was calculated by employing the Chronic Kidney Disease Epidemiology Collaboration (CKD-EPI) equation [23]. The CKD-EPI equation is as follows: eGFR = 141 × min(Scr/κ, 1)^α × max(Scr/κ, 1)^-1.209 × 0.993^Age × 1.018 [if female] × 1.159 [if Black]. Where: [1] Scr: Serum creatinine concentration [2] κ: 0.7 for females, 0.9 for males [3] α: −0.329 for females, −0.411 for males [4] min: The smaller value of Scr/κ or 1 [5] max: The greater value of Scr/κ or 1. The terms (1.018 if female) and (1.159 if Black) are multiplicative factors applied only if the respective conditions are met. The result is in mL/min/1.73m^2^. The definition of hypertension adopted the 2017 American Heart Association (AHA) hypertension guidelines: a systolic blood pressure reaching or exceeding 130 mmHg, or a diastolic blood pressure reaching or exceeding 80 mmHg, based on the average of three blood pressure measurements. Diabetic patients are defined as those using antidiabetic drugs or insulin to control blood glucose, or having a glycohemoglobin level ≥6.5%. Meeting any one of these criteria qualifies.

### Statistical analysis

This study utilized R software (version 4.4.1) and EmpowerStats software (www.empowerstats.com) for statistical analysis. The significance level was set at *P* < 0.05. In EmpowerStats, we utilized the following modules for analysis: Research Population Description, Univariate Analysis, Generalized Linear Models, Interaction Effect Tests, Smooth Curve Fitting, and Threshold and Saturation Effects Analysis. R software was primarily used for data cleaning, organization, and handling missing data. Packages including “dplyr” and “mice” were utilized. Handling of missing data was performed through multiple imputation using the random forest method. The imputed data accounted for approximately 3% of the total covariates, with detailed post-imputation data presented in S1 File. The weighting factors used were WTSAF2YR/4, SDMVPSU, and SDMVSTRA. After weighting, the participants represented the US population as shown in S2 File. Continuous data and categorical data were presented as mean (standard deviation) and number (proportion), respectively. Group comparisons were made using a Student’s test and a chi-squared test for continuous and categorical data. The association between AIP and the odds of HUA was investigated through a multivariate logistic regression model. Model 1 made no adjustments for other covariates; Model 2 was adjusted for age, sex, and race; Model 3 was adjusted for all covariates [24]. The presence of nonlinear associations was assessed through smooth curve fitting and generalized additive models. The recursive method was employed to determine the location of the inflection point and establish a two-segment linear regression model on both sides. Subgroup analysis was performed using stratified multivariate regression analysis. To prevent overfitting, ensure that the effective degrees of freedom remain sufficiently low—specifically, they are much smaller than the sample size, using thresholds such as effective degrees of freedom < sample size/10 or more conservative criteria. Additionally, apply False Discovery Rate (FDR) correction to the p-values for subgroup interactions.

## Results

### Baseline characteristics

Tables 1 and 2, respectively, presented the baseline characteristics of the study population according to HUA status and AIP quartiles. The average age of the participants in this study was 45.33 ± 16.45 years, with 47.16% of the participants being male and 52.84% being female. Regarding ethnic distribution, the majority of participants were Non-Hispanic White (35.96%), followed by Non-Hispanic Black (21.26%), individuals of other races (17.39%), Mexican American (14.43%), and finally, Other Hispanic (10.97%). The overall prevalence of HUA was found to be 19.12%. Participants with HUA are often associated with older age, better financial status, obesity, heavy alcohol consumption, insufficient physical activity, hypertension, and diabetes. In the AIP quartile grouping, male patients with obesity, poverty, smoking habits, heavy alcohol consumption, physical inactivity, hypertension, and diabetes, as well as poorer laboratory results (such as lower eGFR, elevated aspartate aminotransferase, alanine aminotransferase, and LDL-C levels), tend to exhibit elevated AIP levels (Table 3).

**Table 1:** Baseline characteristics of participants according to HUA status.

Characteristic	Number of Participants			*P*-value
	Total (N = 7057)	Non-HUA (N = 5708)	HUA (N = 1349)	
Age (years)	45.33 ± 16.45	44.75 ± 16.19	47.77 ± 17.29	<0.001
Gender, n (%)				0.27
Male	3328 (47.16%)	2710 (47.48%)	618 (45.81%)	
Female	3729 (52.84%)	2998 (52.52%)	731 (54.19%)	
Race, n (%)				<0.001
Mexican American	1018 (14.43%)	867 (15.19%)	151 (11.19%)	
Other Hispanic	774 (10.97%)	651 (11.41%)	123 (9.12%)	
Non-Hispanic White	2538 (35.96%)	2041 (35.76%)	497 (36.84%)	
Non-Hispanic Black	1500 (21.26%)	1155 (20.23%)	345 (25.57%)	
Other Race	1227 (17.39%)	994 (17.41%)	233 (17.27%)	
PIR	2.45 ± 1.64	2.43 ± 1.63	2.54 ± 1.64	0.017
Education, n (%)				0.002
Less than 9th grade	597 (8.46%)	505 (8.85%)	92 (6.82%)	
9-11th grade	921 (13.05%)	763 (13.37%)	158 (11.71%)	
High school graduate	1506 (21.34%)	1199 (21.01%)	307 (22.76%)	
Some college or AA degree	2164 (30.66%)	1706 (29.89%)	458 (33.95%)	
College graduate or above	1869 (26.48%)	1535 (26.89%)	334 (24.76%)	
BMI,n (%)				<0.001
Normal weight	2270 (32.17%)	2057 (36.04%)	213 (15.79%)	
Overweight	2239 (31.73%)	1866 (32.69%)	373 (27.65%)	
Obesity	2548 (36.11%)	1785 (31.27%)	763 (56.56%)	
Waist circumference (cm)	97.75 ± 16.66	95.52 ± 15.51	107.18 ± 18.00	<0.001
Aspartate Aminotransferase (U/L)	24.52 ± 20.33	23.95 ± 20.78	26.93 ± 18.11	<0.001
Alanine Aminotransferase (U/L)	24.25 ± 18.11	23.30 ± 16.82	28.29 ± 22.31	<0.001
Serum Creatinine (mg/dL)	0.85 ± 0.34	0.83 ± 0.32	0.94 ± 0.39	<0.001
eGFR (mL/min /1.73m2)	99.73 ± 21.32	101.59 ± 20.07	91.83 ± 24.40	<0.001
Blood Urea Nitrogen (mg/dL)	13.04 ± 4.85	12.68 ± 4.34	14.57 ± 6.37	<0.001
LDL-C (mmol/L)	2.99 ± 0.90	2.97 ± 0.89	3.10 ± 0.95	<0.001
Total Cholesterol (mmol/L)	4.97 ± 1.03	4.93 ± 1.02	5.11 ± 1.08	<0.001
Smoking status, n (%)				<0.001
No smoking	4174 (59.15%)	3402 (59.60%)	772 (57.23%)	
Quit smoking	1415 (20.05%)	1089 (19.08%)	326 (24.17%)	
Current smoking	1468 (20.80%)	1217 (21.32%)	251 (18.61%)	
Drinking status, n (%)				0.014
Low drinking	6094 (86.35%)	4957 (86.84%)	1137 (84.28%)	
Heavy drinking	963 (13.65%)	751 (13.16%)	212 (15.72%)	
Physical Activity, n (%)				<0.001
Insuffciently active	2951 (41.82%)	2321 (40.66%)	630 (46.70%)	
Active	4106 (58.18%)	3387 (59.34%)	719 (53.30%)	
Hypertension, n (%)				<0.001
No	4744 (67.22%)	3954 (69.27%)	790 (58.56%)	
Yes	2313 (32.78%)	1754 (30.73%)	559 (41.44%)	
Diabetes, n (%)				<0.001
No	6397 (90.65%)	5224 (91.52%)	1173 (86.95%)	
Yes	660 (9.35%)	484 (8.48%)	176 (13.05%)	
Serum Uric Acid (mg/dL)	5.37 ± 1.37	4.94 ± 1.03	7.21 ± 1.07	<0.001
Triglyceride (mmol/L)	1.24 ± 0.80	1.17 ± 0.75	1.53 ± 0.92	<0.001
HDL-C (mmol/L)	1.41 ± 0.42	1.43 ± 0.42	1.32 ± 0.40	<0.001
AIP	−0.11 ± 0.32	−0.14 ± 0.32	0.01 ± 0.31	<0.001

Data are presented as number (%) or mean ± standard deviation.

AIP, atherogenic index of plasma; PIR, ratio of family income to poverty; BMI, body.

mass index; eGFR, estimated glomerular filtration rate; HDL-C, high-density lipoprotein cholesterol; LDL-C, low-density lipoprotein cholesterol.

**Table 2:** Baseline characteristics of participants according to AIP quartiles.

Characteristic	AIP				*P*-value
	Q1 (<−0.34)	Q2 (−0.34 to < −0.12)	Q3 (−0.12 to < 0.10)	Q4 (>0.10)	
Age (years)	43.75 ± 17.25	45.23 ± 16.87	46.49 ± 16.47	45.84 ± 15.00	<0.001
Gender, n (%)					<0.001
Male	594 (33.67%)	749 (42.46%)	888 (50.34%)	1097 (62.15%)	
Female	1170 (66.33%)	1015 (57.54%)	876 (49.66%)	668 (37.85%)	
Race, n (%)					<0.001
Mexican American	161 (9.13%)	223 (12.64%)	297 (16.84%)	337 (19.09%)	
Other Hispanic	146 (8.28%)	167 (9.47%)	229 (12.98%)	232 (13.14%)	
Non-Hispanic White	607 (34.41%)	613 (34.75%)	628 (35.60%)	690 (39.09%)	
Non-Hispanic Black	531 (30.10%)	457 (25.91%)	315 (17.86%)	197 (11.16%)	
Other Race	319 (18.08%)	304 (17.23%)	295 (16.72%)	309 (17.51%)	
PIR	2.61 ± 1.67	2.51 ± 1.64	2.36 ± 1.63	2.31 ± 1.59	<0.001
Education, n (%)					<0.001
Less than 9th grade	103 (5.84%)	139 (7.88%)	160 (9.07%)	195 (11.05%)	
9-11th grade	183 (10.37%)	192 (10.88%)	257 (14.57%)	289 (16.37%)	
High school graduate	331 (18.76%)	380 (21.54%)	417 (23.64%)	378 (21.42%)	
Some college or AA degree	571 (32.37%)	556 (31.52%)	515 (29.20%)	522 (29.58%)	
College graduate or above	576 (32.65%)	497 (28.17%)	415 (23.53%)	381 (21.59%)	
BMI,n (%)					<0.001
Normal weight	922 (52.27%)	655 (37.13%)	426 (24.15%)	267 (15.13%)	
Overweight	474 (26.87%)	543 (30.78%)	600 (34.01%)	622 (35.24%)	
Obesity	368 (20.86%)	566 (32.09%)	738 (41.84%)	876 (49.63%)	
Waist circumference (cm)	89.69 ± 15.15	95.96 ± 16.44	100.62 ± 15.66	104.73 ± 15.50	<0.001
Aspartate Aminotransferase (U/L)	23.52 ± 16.43	23.87 ± 25.01	24.21 ± 13.73	26.48 ± 23.76	<0.001
Alanine Aminotransferase (U/L)	20.04 ± 15.47	22.02 ± 18.70	24.84 ± 16.15	30.10 ± 20.12	<0.001
Serum Creatinine (mg/dL)	0.82 ± 0.34	0.84 ± 0.28	0.86 ± 0.37	0.88 ± 0.36	<0.001
eGFR (mL/min /1.73m2)	102.53 ± 21.52	100.07 ± 21.28	98.29 ± 20.80	98.02 ± 21.38	<0.001
Blood Urea Nitrogen (mg/dL)	12.84 ± 4.58	12.91 ± 4.71	13.04 ± 4.82	13.38 ± 5.24	0.005
LDL-C (mmol/L)	2.64 ± 0.79	2.94 ± 0.80	3.16 ± 0.92	3.23 ± 0.97	<0.001
Total Cholesterol (mmol/L)	4.72 ± 0.96	4.83 ± 0.94	5.01 ± 1.05	5.31 ± 1.08	<0.001
Smoking status, n (%)					<0.001
No smoking	1147 (65.02%)	1104 (62.59%)	1015 (57.54%)	908 (51.44%)	
Quit smoking	338 (19.16%)	331 (18.76%)	368 (20.86%)	378 (21.42%)	
Current smoking	279 (15.82%)	329 (18.65%)	381 (21.60%)	479 (27.14%)	
Drinking status, n (%)					<0.001
Low drinking	1575 (89.29%)	1536 (87.07%)	1505 (85.32%)	1478 (83.74%)	
Heavy drinking	189 (10.71%)	228 (12.93%)	259 (14.68%)	287 (16.26%)	
Physical Activity, n (%)					<0.001
Insuffciently active	655 (37.13%)	705 (39.97%)	793 (44.95%)	798 (45.21%)	
Active	1109 (62.87%)	1059 (60.03%)	971 (55.05%)	967 (54.79%)	
Hypertension, n (%)					<0.001
No	1306 (74.04%)	1242 (70.41%)	1118 (63.38%)	1078 (61.08%)	
Yes	458 (25.96%)	522 (29.59%)	646 (36.62%)	687 (38.92%)	
Diabetes, n (%)					<0.001
No	1686 (95.58%)	1643 (93.14%)	1573 (89.17%)	1495 (84.70%)	
Yes	78 (4.42%)	121 (6.86%)	191 (10.83%)	270 (15.30%)	
Triglyceride (mmol/L)	0.57 ± 0.16	0.88 ± 0.19	1.24 ± 0.26	2.27 ± 0.89	<0.001
HDL-C (mmol/L)	1.82 ± 0.44	1.48 ± 0.29	1.28 ± 0.24	1.05 ± 0.20	<0.001
Serum uric acid (mg/dL)	4.82 ± 1.19	5.18 ± 1.24	5.53 ± 1.34	5.96 ± 1.41	<0.001
Hyperuricemia, n (%)					<0.001
No	1586 (89.91%)	1491 (84.52%)	1381 (78.29%)	1250 (70.82%)	
Yes	178 (10.09%)	273 (15.48%)	383 (21.71%)	515 (29.18%)	

Data are presented as number (%) or mean ± standard deviation.

AIP, atherogenic index of plasma; PIR, ratio of family income to poverty; BMI, body

mass index; eGFR, estimated glomerular filtration rate; HDL-C, high-density lipoprotein cholesterol; LDL-C, low-density lipoprotein cholesterol

**Table 3:** The association between AIP and hyperuricemia.

	Model 1 OR (95% CI)	Model 2 OR (95% CI)	Model 3 OR (95% CI)
**AIP**			
Per 1 increment	4.37 (3.62, 5.28)***	5.95 (4.86, 7.29)***	3.22 (2.54, 4.10)***
Q1	Reference	Reference	Reference
Q2	1.63 (1.33, 2.00)***	1.72 (1.41, 2.11)***	1.48 (1.18, 1.85)***
Q3	2.47 (2.04, 2.99)***	2.85 (2.34, 3.46)***	2.17 (1.74, 2.71)***
Q4	3.67 (3.05, 4.42)***	4.65 (3.82, 5.65)***	2.76 (2.20, 3.45)***
P for trend	<0.0001	<0.0001	<0.0001
**TG**			
Per 1 increment	1.60 (1.50, 1.71)***	1.71 (1.60, 1.84)***	1.49 (1.36, 1.64)***
Q1	Reference	Reference	Reference
Q2	1.90 (1.55, 2.32)***	1.98 (1.62, 2.44)***	1.67 (1.34, 2.08)***
Q3	2.54 (2.09, 3.09)***	2.81 (2.30, 3.44)***	2.19 (1.76, 2.73)***
Q4	3.65 (3.02, 4.41)***	4.35 (3.57, 5.31)***	2.73 (2.17, 3.45)***
P for trend	<0.0001	<0.0001	<0.0001
**HDL-C**			
Per 1 increment	0.48 (0.40, 0.56)***	0.36 (0.30, 0.43)***	0.41 (0.33, 0.52)***
Q1	Reference	Reference	Reference
Q2	0.71 (0.61, 0.83)***	0.64 (0.55, 0.76)***	0.73 (0.61, 0.87)***
Q3	0.56 (0.47, 0.66)***	0.47 (0.39, 0.55)***	0.60 (0.49, 0.73)***
Q4	0.43 (0.36, 0.51)***	0.32 (0.27, 0.39)***	0.41 (0.32, 0.52)***
P for trend	<0.0001	<0.0001	<0.0001

Data are presented as OR (95% CI)

AIP, atherogenic index of plasma; TG, triglyceride; HDL-C, high-density lipoprotein cholesterol

*P < 0.05

**P < 0.01

***P < 0.001

Model 1: No covariates were adjusted

Model 2: Age, gender, and race were adjusted

Model 3: Age, gender, race, PIR, education, BMI, waist circumference, aspartate aminotransferase, alanine aminotransferase, serum creatinine, eGFR, blood urea nitrogen, total cholesterol, LDL-C, smoking status, drinking status, physical activity, hypertension, and diabetes were adjusted

### Association of the AIP and hyperuricemia

**Fig 2:**
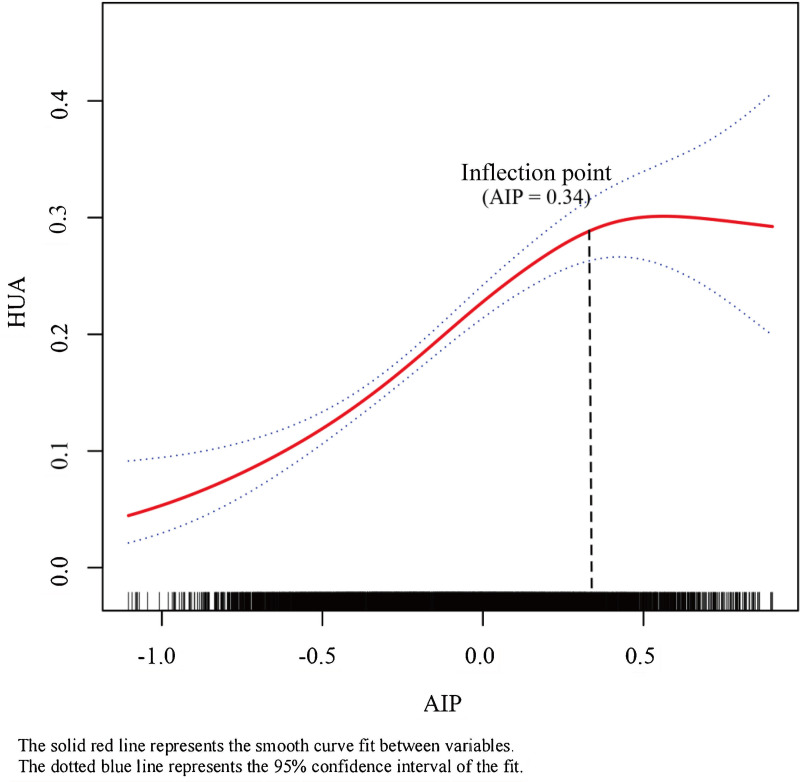
Smooth curve fitting of the relationship between the AIP and hyperuricemia. Age, gender, race, PIR, education, BMI, waist circumference, aspartate aminotransferase, alanine aminotransferase, serum creatinine, eGFR, blood urea nitrogen, total cholesterol, LDL-C, smoking status, drinking status, physical activity, hypertension, and diabetes were adjusted.

Across all three models, a significant positive association was demonstrated between AIP and the risk of HUA: Model 1 (OR = 4.37, 95%CI [3.62, 5.28], P < 0.001); Model 2 (OR = 5.95, 95%CI [4.86, 7.29], P < 0.001); Model 3 (OR = 3.22, 95%CI [2.54, 4.10], P < 0.001). In Model 3, the top AIP quartile (Q4) (OR = 2.76, 95%CI [2.20, 3.45], P < 0.001) had a 1.76-fold higher risk of HUA than the reference AIP quartile (Q1). The P-value for the trend test was less than 0.0001. The relationships of TG and HDL-C within the AIP formula with HUA were also presented in Table 3. TG showed a significant positive correlation with HUA, while HDL-C showed a significant negative correlation with HUA. Stratified analyses were performed in two key subgroups (gender subgroups and BMI subgroups), with detailed analysis contents as shown in Table 4. A significant positive correlation remains consistent between AIP and the risk of HUA among males, females, and participants with normal weight, overweight, and obesity. Meanwhile, the study revealed a non-linear association between AIP and the risk of HUA, which manifests as an inverted L-shaped curve with an inflection point located at 0.34, as shown in Fig 2. Before the inflection point of 0.34, there was a positive correlation between AIP and the risk of HUA. For each 1 unit increment in AIP, the risk of HUA increases by a factor of 3.37. (OR = 4.37, 95%CI [3.27, 5.85], P < 0.001). After reaching the inflection point of 0.34, the correlation between AIP and the odds of HUA was no longer statistically significant (OR = 0.46, 95%CI [0.16, 1.35], P = 0.158) (Table 5).

**Table 4:** The association between AIP and hyperuricemia stratified by gender and BMI.

	Model 1 OR (95% CI)	Model 2 OR (95% CI)	Model 3 OR (95% CI)
**Male**			
Per 1 increment	3.56 (2.71, 4.68)***	4.21 (3.18, 5.58)***	2.24 (1.59, 3.16)***
Q1	Reference	Reference	Reference
Q2	1.76 (1.24, 2.49)**	1.85 (1.30, 2.62)***	1.59 (1.10, 2.30)*
Q3	2.65 (1.91, 3.67)***	2.95 (2.12, 4.10)***	2.19 (1.53, 3.13)***
Q4	3.37 (2.46, 4.60)***	3.92 (2.84, 5.40)***	2.24 (1.57, 3.19)***
P for trend	<0.0001	<0.0001	<0.0001
**Female**			
Per 1 increment	6.75 (5.11, 8.91)***	8.19 (6.10, 10.99)***	4.45 (3.13, 6.31)***
Q1	Reference	Reference	Reference
Q2	1.60 (1.25, 2.05)***	1.63 (1.27, 2.10)***	1.35 (1.02, 1.79)*
Q3	2.47 (1.94, 3.16)***	2.64 (2.06, 3.40)***	1.95 (1.46, 2.61)***
Q4	4.78 (3.75, 6.10)***	5.50 (4.26, 7.09)***	3.26 (2.42, 4.40)***
P for trend	<0.0001	<0.0001	<0.0001
**Normal weight**			
Per 1 increment	4.91 (3.08, 7.81)***	4.80 (2.94, 7.84)***	4.73 (2.73, 8.19)***
Q1	Reference	Reference	Reference
Q2	1.67 (1.12, 2.48)*	1.67 (1.11, 2.50)*	2.09 (1.34, 3.25)**
Q3	2.75 (1.84, 4.11)***	2.82 (1.86, 4.27)***	3.56 (2.22, 5.71)***
Q4	4.21 (2.77, 6.40)***	4.19 (2.69, 6.51)***	4.49 (2.69, 7.52)***
P for trend	<0.0001	<0.0001	<0.0001
**Overweight**			
Per 1 increment	3.33 (2.34, 4.74)***	4.28 (2.91, 6.28)***	3.52 (2.30, 5.38)***
Q1	Reference	Reference	Reference
Q2	1.35 (0.93, 1.96)	1.41 (0.97, 2.06)	1.52 (1.02, 2.27)*
Q3	1.42 (0.99, 2.04)	1.56 (1.07, 2.26)*	1.56 (1.04, 2.33)*
Q4	2.52 (1.80, 3.55)***	3.00 (2.09, 4.30)***	2.68 (1.81, 3.98)***
P for trend	<0.0001	<0.0001	<0.0001
**Obesity**			
Per 1 increment	2.44 (1.84, 3.22)***	3.15 (2.32, 4.28)***	2.95 (2.06, 4.22)***
Q1	Reference	Reference	Reference
Q2	1.24 (0.90, 1.70)	1.30 (0.95, 1.80)	1.16 (0.82, 1.64)
Q3	1.80 (1.33, 2.41***	2.07 (1.52, 2.81)***	2.00 (1.43, 2.79)***
Q4	2.15 (1.61, 2.86)***	2.64 (1.94, 3.59)***	2.26 (1.61, 3.16)***
P for trend	<0.0001	<0.0001	<0.0001

Data are presented as OR (95% CI)

*P < 0.05

**P < 0.01

***P < 0.001

Model 1: No covariates were adjusted

Model 2: Age, gender, and race were adjusted, except for the variable itself

Model 3: Age, gender, race, PIR, education, BMI, waist circumference, aspartate aminotransferase, alanine aminotransferase, serum creatinine, eGFR, blood urea nitrogen, total cholesterol, LDL-C, smoking status, drinking status, physical activity, hypertension, and diabetes were adjusted, except for the variable itself

**Table 5:** Threshold effect analysis of AIP on hyperuricemia.

Serum uric acid	Adjusted OR (95% CI)	P-value
Fitting by the standard linear model	3.22 (2.54, 4.10)	<0.001
Fitting by the two-piecewise linear model		
Infection point	0.34	
AIP < 0.34	4.37 (3.27, 5.85)	<0.001
AIP > 0.34	0.46 (0.16, 1.35)	0.158
Log likelihood ratio test		<0.001

Age, gender, race, PIR, education, BMI, waist circumference, aspartate

aminotransferase, alanine aminotransferase, serum creatinine, eGFR, blood urea nitrogen, total cholesterol, LDL-C, smoking status, drinking status, physical activity, hypertension, and diabetes were adjusted

### Subgroup analysis

Subgroup analyses were conducted based on age, gender, race, educational background, BMI, smoking status, drinking status, physical activity, renal function, hypertension, and diabetes, with corresponding P values for interaction calculated.

**Fig 3:**
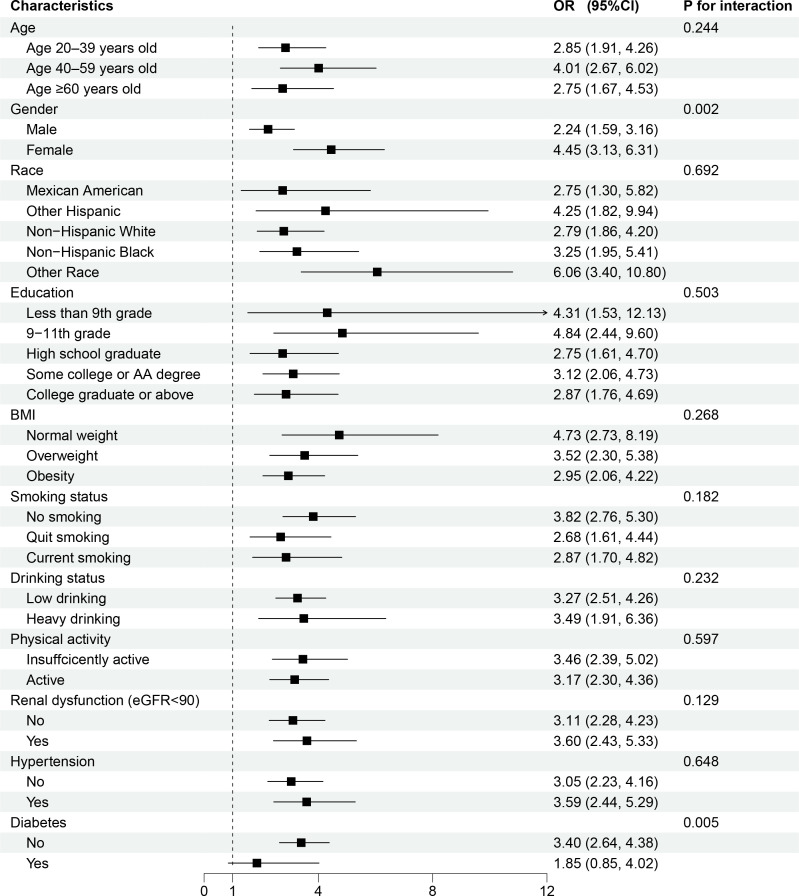
Forest plots of subgroup analyses for the association between AIP and hyperuricemia. Age, gender, race, PIR, education, BMI, waist circumference, aspartate. Aminotransferase, alanine aminotransferase, serum creatinine, eGFR, blood urea nitrogen, total cholesterol, LDL-C, smoking status, drinking status, physical activity, hypertension, and diabetes were adjusted, except for the variable itself.

Age and eGFR, originally continuous variables, were converted into categorical variables for analysis based on thresholds of 20–39, 40–59, and ≥60 years and 90 mL/min/1.73 m². The detailed results are presented in a table and a forest plot, as shown in Table 6 and Fig 3. Except for the non-significant association between AIP and the risk of HUA in diabetic population (OR = 1.85, 95%CI [0.85, 4.02], P = 0.123), a consistent and statistically significant positive correlation of AIP with HUA was observed in all subgroups (including age, gender, race, educational background, BMI, smoking status, drinking status, physical activity, renal function, hypertension). The p-values for the interactions in age, race, educational background, BMI, smoking status, drinking status, physical activity, renal function, and hypertension, subgroups were all found to be greater than 0.05, showing no statistical significance. However, for the gender subgroup, the interaction p-value was 0.002, which is less than 0.05. This indicates that the association between AIP and the risk of HUA different significantly between males and females, with females exhibiting a higher risk than males.

**Table 6:** Subgroup analysis for the association between AIP and hyperuricemia.

	Number of sample	OR (95% CI)	P-value	P for interaction
**Stratified by age**				0.244
Age 20–39 years old	2928	2.85 (1.91, 4.26)	<0.001	
Age 40–59 years old	2532	4.01 (2.67, 6.02)	<0.001	
Age ≥ 60 years old	1597	2.75 (1.67, 4.53)	<0.001	
**Stratified by gender**				0.002
Male	3328	2.24 (1.59, 3.16)	<0.001	
Female	3729	4.45 (3.13, 6.31)	<0.001	
**Stratified by race**				0.692
Mexican American	1018	2.75 (1.30, 5.82)	0.008	
Other Hispanic	774	4.25 (1.82, 9.94)	<0.001	
Non-Hispanic White	2538	2.79 (1.86, 4.20)	<0.001	
Non-Hispanic Black	1500	3.25 (1.95, 5.41)	<0.001	
Other Race	1227	6.06 (3.40, 10.80)	<0.001	
**Stratified by education**				0.503
Less than 9th grade	597	4.31 (1.53, 12.13)	0.006	
9-11th grade	921	4.84 (2.44, 9.60)	<0.001	
High school graduate	1506	2.75 (1.61, 4.70)	<0.001	
Some college or AA degree	2164	3.12 (2.06, 4.73)	<0.001	
College graduate or above	1869	2.87 (1.76, 4.69)	<0.001	
**Stratified by BMI**				0.268
Normal weight	2270	4.73 (2.73, 8.19)	<0.001	
Overweight	2239	3.52 (2.30, 5.38)	<0.001	
Obesity	2548	2.95 (2.06, 4.22)	<0.001	
**Stratified by smoking status**				0.182
No smoking	4174	3.82 (2.76, 5.30)	<0.001	
Quit smoking	1415	2.68 (1.61, 4.44)	<0.001	
Current smoking	1468	2.87 (1.70, 4.82)	<0.001	
**Stratified by drinking status**				0.232
Low drinking	6094	3.27 (2.51, 4.26)	<0.001	
Heavy drinking	963	3.49 (1.91, 6.36)	<0.001	
**Stratified by physical activity**				0.597
Insuffciently active	2951	3.46 (2.39, 5.02)	<0.001	
Active	4106	3.17 (2.30, 4.36)	<0.001	
**Stratified by renal function**				0.129
eGFR < 90mL/min /1.73m2	2108	3.60 (2.43, 5.33)	<0.001	
eGFR ≥ 90mL/min /1.73m2	4949	3.11 (2.28, 4.23)	<0.001	
**Stratified by hypertension**				0.648
No	4744	3.05 (2.23, 4.16)	<0.001	
Yes	2313	3.59 (2.44, 5.29)	<0.001	
**Stratified by diabetes**				0.005
No	6397	3.40 (2.64, 4.38)	<0.001	
Yes	660	1.85 (0.85, 4.02)	0.123	

Age, gender, race, PIR, education, BMI, waist circumference, aspartate

aminotransferase, alanine aminotransferase, serum creatinine, eGFR, blood urea nitrogen, total cholesterol, LDL-C, smoking status, drinking status, physical activity, hypertension, and diabetes were adjusted, except for the variable itself

## Discussion

Among US adults, AIP and HUA risk demonstrate a significantly positive correlation. This relationship follows an inverted L-shaped curve. Further subgroup analysis revealed that female patients with elevated AIP face a higher risk of developing HUA than their male counterparts.

Compared to previous studies on the association between AIP and the risk of HUA, this research shows both similarities and unique innovative aspects in its conclusions. The positive correlation we found in the U.S. population is consistent with observations in populations from other regions. Xu et al. used data from the China Health and Retirement Longitudinal Study (CHARLS) to investigate the association between the AIP and HUA. Their research results indicated a positive association between AIP and the odds of HUA in the Chinese population. In the fully adjusted model, the group with the highest AIP quartile had a 2.81-fold higher risk of HUA compared to the reference AIP quartile group [25]. A retrospective study on an Italian outpatient population showed that patients with HUA had significantly higher AIP values compared to those with normal SUA levels, and this was observed in both male and female patients [26]. The same conclusion was also drawn among the Chinese population by another cross-sectional study conducted in China [27]. In addition to the aforementioned similarities, our study also yielded findings that differ from previous studies. Firstly, as noted in the introduction, most previous studies on AIP and HUA have focused on non-U.S. populations. This study represents a comprehensive investigation into the relationship between AIP and HUA within the U.S. population, thereby addressing a gap in existing research. Secondly, this study is the first to report a nonlinear inverted L-shaped relationship between AIP and HUA in the US population. Before the turning point of 0.34, there was a significant and rapid rising stage, and after reaching the turning point of 0.34, the calculated OR values fell below 1 and no longer showed statistical significance. The study conducted by Xu et al. is the sole retrieved literature that employed restricted cubic spline analysis to flexibly model and visualize this association in the Chinese population, yet no significant evidence of a nonlinear association was detected, and all curves demonstrated a rising trend in the OR with increasing AIP levels [25]. Thirdly, Similar to Ye et al.‘s findings in the US population, a significantly positive AIP-HUA risk association was demonstrated across both hypertensive and non-hypertensive cohorts in our research [22]. Our study shows that the positive correlation between AIP and HUA remained consistent across all subgroups except the diabetic population. Therefore, when drawing conclusions about the positive association between AIP and the risk of HUA in the US population, it may be necessary to highlight the exclusion of the diabetic population. Additionally, both gender and diabetes had a significant interaction with the AIP in this study, which differed from findings based on the Chinese population. In Xu et al.’s study, consuming alcohol more than once per month exacerbated the association between AIP and HUA (P for interaction = 0.02), while after propensity score matching, smoking status showed a significant interaction with AIP (P for interaction = 0.02) [25]. Overall, our findings show greater similarity to U.S.-based studies and discrepancy from Chinese population studies, underscoring the necessity of our research.

The potential mechanism linking elevated AIP with increased risk of HUA has not been elucidated. From the perspective of TG and HDL, we propose the following possible explanations. (Ⅰ) UA influences TG metabolism. After instilling a saturated UA solution into the bladders of rats, their serum TG concentration was significantly higher than that of the control group instilled with saline [28]. Another study found that hepatic lipase activity was lower in the group of mice with high SUA compared to the low SUA group, resulting in elevated serum TG levels [29]. (Ⅱ) HDL affects renal function, thereby influencing UA metabolism. Low serum HDL-C levels can lead to decreased GFR and are further associated with the progression of chronic kidney disease [30]. This view has been substantiated by results from in vitro and animal studies [31]. The decline in renal function causes elevated SUA levels. (Ⅲ) Diet and genetic factors. A low-fat diet can help reduce SUA levels, while genetic factors may contribute to the simultaneous occurrence of HUA and hyperlipidemia [32,33]. The question of why an inflection point was not observed in other populations warrants attention. The emergence of such a point appears to be population-specific, as evidenced by its absence in the Chinese cohort mentioned above. Furthermore, prior research has mainly identified an association between AIP and HUA, with limited investigation into potential nonlinear relationships. Additionally,our study did not exclude antacid users, which may have led to an underestimation of HUA prevalence, particularly among high-risk individuals. Given the cross-sectional design adopted here, no causal inferences can be drawn from the results. The reverse L-shaped relationship between AIP and HUA risk requires further investigation to clarify its underlying mechanisms. Future studies should not only address the aforementioned issues but also examine whether later-stage beneficial interventions—such as dietary and exercise management—contribute to reducing the risk of HUA. The AIP exhibits a stronger positive link to HUA risk in women versus men, potentially mediated by estrogen. HUA in women predominantly occurs after menopause. Estrogen can enhance UA excretion, and studies indicate that Estrogen Replacement Therapy lowers UA levels in postmenopausal women with HUA [34]. Meanwhile, estrogen has anti-atherosclerotic effects. Postmenopausal women experience changes in lipid metabolism, manifested primarily as elevated TG and decreased HDL-C levels. These alterations in the lipid profile are linked to reduced estrogen levels [35]. The diabetes subgroup showed no significant correlation in this study may be explained by SUA and blood glucose levels. A UK study revealed that serum glucose levels below approximately 8.0 mmol/L exhibit a positive correlation with SUA concentration; however, when blood glucose levels surpass this threshold, SUA concentration begins to decrease instead. Patients with insulin-dependent diabetes and oral hypoglycemia, as well as “non-diabetics” with temporary blood sugar levels greater than 10 mmol/l, had significantly lower UA levels [36]. A similar pattern was observed in a Chinese study: individuals with impaired glucose regulation had the highest SUA levels, followed by those with normal glucose tolerance, while type 2 diabetes mellitus patients exhibited the lowest levels [37]. One possible cause for those phenomena may be that glucose and UA are reabsorbed via competing cotransporters in the renal proximal tubule. When blood glucose reaches the renal threshold, glucose reabsorption becomes dominant. Since UA cannot be fully reabsorbed, increased urinary UA excretion leads to decreased SUA levels.

This study may offer new perspectives for the public health policies and clinical prevention strategies regarding HUA in the United States. At the public health level, the AIP can serve as an effective biomarker for identifying high-risk HUA populations among the majority of US adults. It simultaneously provides a preliminary screening tool for HUA derived from routine lipid panel tests, thereby facilitating large-scale screening. At the clinical practice level, AIP triggers the interconnection among dyslipidemia, CVD, and HUA. When seeing patients, particularly those with CVD risk factors such as obesity, metabolic syndrome, and hypertension, physicians should calculate their AIP. If the AIP is significantly elevated, clinicians should be vigilant about the patient’s existing or future risk of developing HUA.

This study has the following limitations. First, data on some covariates were obtained through participant questionnaires, which may introduce subjective bias into the study results. Second, as a cross-sectional study, its ability to establish causal relationships is limited, primarily providing evidence of associations. The identified inflection point (AIP = 0.34) should be considered an observational finding that defines a threshold for risk plateau in this population; its predictive value and potential as a clinical target require investigation in longitudinal studies. Third, the study did not exclude patients taking urate-lowering drugs, which may lead to an underestimation of the prevalence of HUA patients, thereby affecting the outcomes. Fourth, the data in this study came exclusively from the NHANES, necessitating further validation through research from other institutions or hospitals to confirm the generalizability of the conclusions.

## Conclusion

Conclusions: AIP and the risk of HUA demonstrated an inverted L-shaped positive association in the adult US population. The association was stronger in females than in males.

## Supporting information

S1 File Supplementary Material 1. (XLSX)

S2 File Supplementary Material 2. (DOCX)

S3 File Supplementary Material 3. (DOCX)
