# Supplementary material for: The relationship between the atherogenic index of plasma and hyperuricemia in American adults aged over 20 years: A cross-sectional study
Source: PLoS One. 2026 Mar 19;21(3):e0344977. doi: 10.1371/journal.pone.0344977 (PMC13001944; doi:10.1371/journal.pone.0344977)
Supplement: S3 File — (DOCX) [file pone.0344977.s003.docx]

No specific accession numbers are needed to access the data. Detailed dataset titles and URLs are as follows.

**2011-2012:**

Demographic Variables & Sample Weights (DEMO_G)

<https://wwwn.cdc.gov/Nchs/Data/Nhanes/Public/2011/DataFiles/DEMO_G.htm>

Body Measures (BMX_G)

<https://wwwn.cdc.gov/Nchs/Data/Nhanes/Public/2011/DataFiles/BMX_G.htm#BMXBMI>

Blood Pressure (BPX_G)

<https://wwwn.cdc.gov/Nchs/Data/Nhanes/Public/2011/DataFiles/BPX_G.htm#BPXSY1>

Cholesterol - HDL (HDL_G)

<https://wwwn.cdc.gov/Nchs/Data/Nhanes/Public/2011/DataFiles/HDL_G.htm>

Cholesterol - LDL & Triglycerides (TRIGLY_G)

<https://wwwn.cdc.gov/Nchs/Data/Nhanes/Public/2011/DataFiles/TRIGLY_G.htm>

Cholesterol - Total (TCHOL_G)

<https://wwwn.cdc.gov/Nchs/Data/Nhanes/Public/2011/DataFiles/TCHOL_G.htm>

Glycohemoglobin (GHB_G)

<https://wwwn.cdc.gov/Nchs/Data/Nhanes/Public/2011/DataFiles/GHB_G.htm>

Standard Biochemistry Profile (BIOPRO_G)

<https://wwwn.cdc.gov/Nchs/Data/Nhanes/Public/2011/DataFiles/BIOPRO_G.htm>

Alcohol Use (ALQ_G)

<https://wwwn.cdc.gov/Nchs/Data/Nhanes/Public/2011/DataFiles/ALQ_G.htm>

Blood Pressure & Cholesterol (BPQ_G)

<https://wwwn.cdc.gov/Nchs/Data/Nhanes/Public/2011/DataFiles/BPQ_G.htm>

Diabetes (DIQ_G)

<https://wwwn.cdc.gov/Nchs/Data/Nhanes/Public/2011/DataFiles/DIQ_G.htm>

Medical Conditions (MCQ_G)

<https://wwwn.cdc.gov/Nchs/Data/Nhanes/Public/2011/DataFiles/MCQ_G.htm>

Physical Activity (PAQ_G)

<https://wwwn.cdc.gov/Nchs/Data/Nhanes/Public/2011/DataFiles/PAQ_G.htm>

Smoking - Cigarette Use (SMQ_G)

<https://wwwn.cdc.gov/Nchs/Data/Nhanes/Public/2011/DataFiles/SMQ_G.htm>

**2013-2014:**

Demographic Variables and Sample Weights (DEMO_H)

<https://wwwn.cdc.gov/Nchs/Data/Nhanes/Public/2013/DataFiles/DEMO_H.htm>

Body Measures (BMX_H)

<https://wwwn.cdc.gov/Nchs/Data/Nhanes/Public/2013/DataFiles/BMX_H.htm>

Blood Pressure (BPX_H)

<https://wwwn.cdc.gov/Nchs/Data/Nhanes/Public/2013/DataFiles/BPX_H.htm>

Cholesterol - HDL (HDL_H)

<https://wwwn.cdc.gov/Nchs/Data/Nhanes/Public/2013/DataFiles/HDL_H.htm>

Cholesterol - LDL & Triglycerides (TRIGLY_H)

<https://wwwn.cdc.gov/Nchs/Data/Nhanes/Public/2013/DataFiles/TRIGLY_H.htm>

Cholesterol - Total (TCHOL_H)

<https://wwwn.cdc.gov/Nchs/Data/Nhanes/Public/2013/DataFiles/TCHOL_H.htm>

Glycohemoglobin (GHB_H)

<https://wwwn.cdc.gov/Nchs/Data/Nhanes/Public/2013/DataFiles/GHB_H.htm>

Standard Biochemistry Profile (BIOPRO_H)

<https://wwwn.cdc.gov/Nchs/Data/Nhanes/Public/2013/DataFiles/BIOPRO_H.htm>

Alcohol Use (ALQ_H)

<https://wwwn.cdc.gov/Nchs/Data/Nhanes/Public/2013/DataFiles/ALQ_H.htm>

Blood Pressure & Cholesterol (BPQ_H)

<https://wwwn.cdc.gov/Nchs/Data/Nhanes/Public/2013/DataFiles/BPQ_H.htm>

Diabetes (DIQ_H)

https://wwwn.cdc.gov/Nchs/Data/Nhanes/Public/2013/DataFiles/DIQ_H.htm

Medical Conditions (MCQ_H)

<https://wwwn.cdc.gov/Nchs/Data/Nhanes/Public/2013/DataFiles/MCQ_H.htm>

Physical Activity (PAQ_H)

<https://wwwn.cdc.gov/Nchs/Data/Nhanes/Public/2013/DataFiles/PAQ_H.htm>

Smoking - Cigarette Use (SMQ_H)

<https://wwwn.cdc.gov/Nchs/Data/Nhanes/Public/2013/DataFiles/SMQ_H.htm>

**2015-2016:**

Demographic Variables and Sample Weights (DEMO_I)

<https://wwwn.cdc.gov/Nchs/Data/Nhanes/Public/2015/DataFiles/DEMO_I.htm>

Body Measures (BMX_I)

<https://wwwn.cdc.gov/Nchs/Data/Nhanes/Public/2015/DataFiles/BMX_I.htm>

Blood Pressure (BPX_I)

<https://wwwn.cdc.gov/Nchs/Data/Nhanes/Public/2015/DataFiles/BPX_I.htm>

Cholesterol - High-Density Lipoprotein (HDL) (HDL_I)

<https://wwwn.cdc.gov/Nchs/Data/Nhanes/Public/2015/DataFiles/HDL_I.htm>

Cholesterol - Low - Density Lipoprotein (LDL) & Triglycerides (TRIGLY_I)

<https://wwwn.cdc.gov/Nchs/Data/Nhanes/Public/2015/DataFiles/TRIGLY_I.htm>

Cholesterol - Total (TCHOL_I)

<https://wwwn.cdc.gov/Nchs/Data/Nhanes/Public/2015/DataFiles/TCHOL_I.htm>

Glycohemoglobin (GHB_I)

<https://wwwn.cdc.gov/Nchs/Data/Nhanes/Public/2015/DataFiles/GHB_I.htm>

Standard Biochemistry Profile (BIOPRO_I)

<https://wwwn.cdc.gov/Nchs/Data/Nhanes/Public/2015/DataFiles/BIOPRO_I.htm>

Alcohol Use (ALQ_I)

<https://wwwn.cdc.gov/Nchs/Data/Nhanes/Public/2015/DataFiles/ALQ_I.htm>

Blood Pressure & Cholesterol (BPQ_I)

<https://wwwn.cdc.gov/Nchs/Data/Nhanes/Public/2015/DataFiles/BPQ_I.htm>

Diabetes (DIQ_I)

<https://wwwn.cdc.gov/Nchs/Data/Nhanes/Public/2015/DataFiles/DIQ_I.htm>

Medical Conditions (MCQ_I)

<https://wwwn.cdc.gov/Nchs/Data/Nhanes/Public/2015/DataFiles/MCQ_I.htm>

Physical Activity (PAQ_I)

<https://wwwn.cdc.gov/Nchs/Data/Nhanes/Public/2015/DataFiles/PAQ_I.htm>

Smoking - Cigarette Use (SMQ_I)

<https://wwwn.cdc.gov/Nchs/Data/Nhanes/Public/2015/DataFiles/SMQ_I.htm>

**2017-2018:**

Demographic Variables and Sample Weights (DEMO_J)

<https://wwwn.cdc.gov/Nchs/Data/Nhanes/Public/2017/DataFiles/DEMO_J.htm>

Body Measures (BMX_J)

<https://wwwn.cdc.gov/Nchs/Data/Nhanes/Public/2017/DataFiles/BMX_J.htm>

Blood Pressure (BPX_J)

<https://wwwn.cdc.gov/Nchs/Data/Nhanes/Public/2017/DataFiles/BPX_J.htm>

Cholesterol - High - Density Lipoprotein (HDL) (HDL_J)

<https://wwwn.cdc.gov/Nchs/Data/Nhanes/Public/2017/DataFiles/HDL_J.htm>

Cholesterol - Low-Density Lipoproteins (LDL) & Triglycerides (TRIGLY_J)

<https://wwwn.cdc.gov/Nchs/Data/Nhanes/Public/2017/DataFiles/TRIGLY_J.htm>

Cholesterol - Total (TCHOL_J)

<https://wwwn.cdc.gov/Nchs/Data/Nhanes/Public/2017/DataFiles/TCHOL_J.htm>

Glycohemoglobin (GHB_J)

<https://wwwn.cdc.gov/Nchs/Data/Nhanes/Public/2017/DataFiles/GHB_J.htm>

Standard Biochemistry Profile (BIOPRO_J)

<https://wwwn.cdc.gov/Nchs/Data/Nhanes/Public/2017/DataFiles/BIOPRO_J.htm>

Alcohol Use (ALQ_J)

<https://wwwn.cdc.gov/Nchs/Data/Nhanes/Public/2017/DataFiles/ALQ_J.htm>

Blood Pressure & Cholesterol (BPQ_J)

<https://wwwn.cdc.gov/Nchs/Data/Nhanes/Public/2017/DataFiles/BPQ_J.htm>

Diabetes (DIQ_J)

<https://wwwn.cdc.gov/Nchs/Data/Nhanes/Public/2017/DataFiles/DIQ_J.htm#DIQ050>

Medical Conditions (MCQ_J)

<https://wwwn.cdc.gov/Nchs/Data/Nhanes/Public/2017/DataFiles/MCQ_J.htm>

Physical Activity (PAQ_J)

<https://wwwn.cdc.gov/Nchs/Data/Nhanes/Public/2017/DataFiles/PAQ_J.htm>

Smoking - Cigarette Use (SMQ_J)

<https://wwwn.cdc.gov/Nchs/Data/Nhanes/Public/2017/DataFiles/SMQ_J.htm>
