# Supplementary material for: The relationship between the atherogenic index of plasma and hyperuricemia in American adults aged over 20 years: A cross-sectional study
Source: PLoS One. 2026 Mar 19;21(3):e0344977. doi: 10.1371/journal.pone.0344977 (PMC13001944; doi:10.1371/journal.pone.0344977)
Supplement: Supplementary file 3 [file pone.0344977.s002.docx]

Characteristic	Number of represented population				P-value
	Q1 (N=44146379)	Q2 (N=43354390)	Q3 (N=43249074)	Q4 (N=43092170)	
Age (years)	41.98 (40.81, 43.14)	43.35 (42.35, 44.34)	44.28 (43.39, 45.16)	44.44 (43.68, 45.20)	0.0083
Gender, (%)					<0.0001
Male	33.89 (30.86, 37.04)	41.84 (39.03, 44.71)	51.34 (47.93, 54.73)	63.20 (60.44, 65.87)	
Female	66.11 (62.96, 69.14)	58.16 (55.29, 60.97)	48.66 (45.27, 52.07)	36.80 (34.13, 39.56)	
Race, (%)					<0.0001
Mexican American	6.21 (4.64, 8.29)	8.65 (6.76, 11.01)	11.53 (9.02, 14.62)	12.82 (10.34, 15.78)	
Other Hispanic	6.10 (4.61, 8.03)	6.18 (4.70, 8.08)	7.85 (6.37, 9.63)	7.86 (6.18, 9.95)	
Non-Hispanic White	61.29 (56.50, 65.87)	62.13 (57.80, 66.27)	61.95 (57.42, 66.29)	64.62 (60.69, 68.35)	
Non-Hispanic Black	16.91 (13.84, 20.50)	14.20 (11.91, 16.85)	9.54 (7.83, 11.59)	5.94 (4.63, 7.59)	
Other Race	9.49 (7.98, 11.26)	8.83 (7.19, 10.81)	9.12 (7.66, 10.82)	8.76 (7.32, 10.47)	
PIR	3.05 (2.94, 3.16)	2.94 (2.78, 3.10)	2.80 (2.64, 2.95)	2.70 (2.55, 2.85)	<0.0001
Education, (%)					<0.0001
Less than 9th grade	3.48 (2.28, 5.27)	4.35 (3.46, 5.45)	4.70 (3.77, 5.85)	6.88 (5.37, 8.77)	
9-11th grade	7.38 (5.95, 9.11)	7.88 (6.41, 9.66)	11.41 (9.12, 14.19)	12.72 (10.77, 14.96)	
High school graduate	17.65 (14.72, 21.01)	22.01 (18.95, 25.41)	24.64 (21.57, 28.00)	22.58 (19.61, 25.86)	
Some college or AA degree	30.77 (27.24, 34.54)	32.72 (29.48, 36.12)	31.75 (28.85, 34.80)	32.99 (29.73, 36.41)	
College graduate or above	40.73 (36.11, 45.52)	33.04 (29.20, 37.13)	27.49 (23.90, 31.40)	24.83 (21.23, 28.82)	
BMI, (%)					<0.0001
Normal weight	54.42 (50.75, 58.05)	35.20 (30.59, 40.10)	23.37 (20.27, 26.78)	12.76 (10.96, 14.81)	
Overweight	28.14 (25.46, 30.97)	32.92 (29.38, 36.66)	34.60 (31.72, 37.60)	34.41 (31.36, 37.59)	
Obesity	17.44 (14.96, 20.25)	31.88 (27.99, 36.05)	42.03 (38.73, 45.39)	52.83 (49.44, 56.19)	
Waist circumference (cm)	89.26 (88.15, 90.37)	96.50 (95.05, 97.94)	101.02 (99.88, 102.16)	106.48 (105.36, 107.59)	<0.0001
Aspartate Aminotransferase (U/L)	23.66 (22.69, 24.63)	23.57 (22.69, 24.44)	24.05 (23.07, 25.04)	26.34 (25.15, 27.53)	0.0043
Alanine Aminotransferase (U/L)	20.54 (19.29, 21.79)	22.28 (21.42, 23.15)	24.98 (24.01, 25.95)	30.41 (29.33, 31.48)	<0.0001
Serum Creatinine (mg/dL)	0.82 (0.80, 0.83)	0.84 (0.82, 0.85)	0.86 (0.84, 0.87)	0.88 (0.86, 0.90)	<0.0001
eGFR (mL/min /1.73m2)	101.71 (100.13, 103.30)	99.52 (98.17, 100.86)	98.06 (96.78, 99.33)	98.06 (96.76, 99.35)	0.0037
Blood Urea Nitrogen (mg/dL)	13.08 (12.76, 13.39)	12.99 (12.74, 13.25)	13.13 (12.78, 13.47)	13.36 (13.06, 13.66)	0.2534
LDL-C (mmol/L)	2.61 (2.57, 2.66)	2.94 (2.89, 3.00)	3.17 (3.11, 3.23)	3.29 (3.24, 3.34)	<0.0001
Total Cholesterol (mmol/L)	4.71 (4.66, 4.76)	4.82 (4.76, 4.89)	5.03 (4.96, 5.09)	5.37 (5.30, 5.44)	<0.0001
Smoking status, (%)					<0.0001
No smoking	63.13 (59.87, 66.26)	64.79 (61.07, 68.33)	56.32 (53.01, 59.58)	50.88 (47.63, 54.11)	
Quit smoking	22.44 (19.41, 25.80)	18.16 (15.69, 20.91)	22.56 (19.44, 26.02)	23.28 (20.55, 26.26)	
Current smoking	14.43 (12.44, 16.69)	17.06 (14.55, 19.89)	21.12 (18.07, 24.52)	25.84 (23.41, 28.42)	
Drinking status, (%)					0.0028
Low drinking	88.84 (86.31, 90.95)	88.08 (86.16, 89.76)	85.37 (82.51, 87.83)	83.58 (80.97, 85.89)	
Heavy drinking	11.16 (9.05, 13.69)	11.92 (10.24, 13.84)	14.63 (12.17, 17.49)	16.42 (14.11, 19.03)	
Physical Activity, (%)					<0.0001
Insuffciently active	31.37 (28.96, 33.89)	36.01 (32.72, 39.43)	39.48 (36.49, 42.55)	40.68 (37.76, 43.67)	
Active	68.63 (66.11, 71.04)	63.99 (60.57, 67.28)	60.52 (57.45, 63.51)	59.32 (56.33, 62.24)	
Hypertension, (%)					<0.0001
No	79.30 (76.83, 81.58)	74.25 (71.28, 77.02)	69.45 (66.51, 72.24)	62.73 (59.48, 65.87)	
Yes	20.70 (18.42, 23.17)	25.75 (22.98, 28.72)	30.55 (27.76, 33.49)	37.27 (34.13, 40.52)	
Diabetes, (%)					<0.0001
No	97.81 (97.07, 98.37)	95.51 (94.11, 96.58)	93.09 (91.26, 94.56)	87.82 (85.84, 89.57)	
Yes	2.19 (1.63, 2.93)	4.49 (3.42, 5.89)	6.91 (5.44, 8.74)	12.18 (10.43, 14.16)	
Triglyceride (mmol/L)	0.57 (0.56, 0.58)	0.88 (0.86, 0.90)	1.25 (1.23, 1.26)	2.27 (2.20, 2.33)	<0.0001
HDL-C (mmol/L)	1.83 (1.80, 1.86)	1.48 (1.46, 1.50)	1.28 (1.27, 1.30)	1.06 (1.04, 1.07)	<0.0001
Serum uric acid (mg/dL)	4.79 (4.71, 4.87)	5.18 (5.10, 5.26)	5.54 (5.46, 5.63)	6.03 (5.93, 6.13)	<0.0001
Hyperuricemia, (%)					<0.0001

Characteristic	Number of represented population			*P* -value
	Total (N=173842013)	Non-HUA (N=140871461)	HUA (N=32970552)	
Age (years)	43.50 (42.90, 44.10)	43.09 (42.41, 43.77)	45.25 (44.00, 46.51)	0.0042
Gender, (%)				0.6759
Male	47.48 (46.02, 48.94)	47.31 (45.62, 49.00)	48.20 (44.50, 51.92)	
Female	52.52 (51.06, 53.98)	52.69 (51.00, 54.38)	51.80 (48.08, 55.50)	
Race, (%)				0.0001
Mexican American	9.78 (7.91, 12.04)	10.32 (8.33, 12.71)	7.50 (5.55, 10.07)	
Other Hispanic	6.99 (5.67, 8.60)	7.35 (5.89, 9.14)	5.45 (4.20, 7.04)	
Non-Hispanic White	62.49 (58.71, 66.12)	61.66 (57.87, 65.31)	66.04 (61.09, 70.66)	
Non-Hispanic Black	11.68 (9.82, 13.84)	11.36 (9.59, 13.42)	13.05 (10.41, 16.23)	
Other Race	9.05 (7.96, 10.28)	9.31 (8.18, 10.58)	7.97 (6.49, 9.74)	
PIR	2.87 (2.76, 2.98)	2.86 (2.75, 2.97)	2.93 (2.78, 3.09)	0.2595
Education, (%)				0.0179
Less than 9th grade	4.84 (3.99, 5.86)	5.03 (4.15, 6.10)	4.03 (2.76, 5.84)	
9-11th grade	9.83 (8.60, 11.22)	10.01 (8.68, 11.50)	9.08 (7.57, 10.87)	
High school graduate	21.70 (19.78, 23.75)	21.41 (19.34, 23.65)	22.91 (19.79, 26.36)	
Some college or AA degree	32.05 (30.05, 34.11)	31.14 (28.89, 33.47)	35.94 (32.41, 39.63)	
College graduate or above	31.58 (28.50, 34.83)	32.41 (29.24, 35.75)	28.03 (23.98, 32.48)	
BMI, (%)				<0.0001
Normal weight	31.58 (29.44, 33.79)	35.82 (33.45, 38.26)	13.45 (11.21, 16.05)	
Overweight	32.49 (31.06, 33.95)	33.80 (32.32, 35.31)	26.90 (23.51, 30.58)	
Obesity	35.93 (33.85, 38.07)	30.38 (28.19, 32.66)	59.65 (55.93, 63.27)	
Waist circumference (cm)	98.26 (97.44, 99.08)	95.79 (94.94, 96.64)	108.79 (107.39, 110.19)	<0.0001
Aspartate Aminotransferase (U/L)	24.40 (23.92, 24.87)	23.72 (23.21, 24.24)	27.28 (26.10, 28.46)	<0.0001
Alanine Aminotransferase (U/L)	24.52 (24.01, 25.03)	23.22 (22.75, 23.68)	30.10 (28.54, 31.66)	<0.0001
Serum Creatinine (mg/dL)	0.85 (0.84, 0.85)	0.83 (0.82, 0.84)	0.92 (0.90, 0.94)	<0.0001
eGFR (mL/min /1.73m2)	99.35 (98.47, 100.23)	100.94 (100.02, 101.86)	92.55 (90.89, 94.21)	<0.0001
Blood Urea Nitrogen (mg/dL)	13.14 (12.96, 13.32)	12.84 (12.67, 13.01)	14.41 (13.99, 14.83)	<0.0001
LDL-C (mmol/L)	3.00 (2.97, 3.03)	2.97 (2.94, 3.00)	3.14 (3.06, 3.21)	0.0003
Total Cholesterol (mmol/L)	4.98 (4.94, 5.02)	4.94 (4.90, 4.98)	5.14 (5.05, 5.23)	0.0002
Smoking status, (%)				0.0011
No smoking	58.81 (56.86, 60.74)	59.23 (57.00, 61.41)	57.04 (53.34, 60.66)	
Quit smoking	21.61 (20.04, 23.27)	20.48 (18.81, 22.26)	26.44 (23.08, 30.09)	
Current smoking	19.58 (17.91, 21.35)	20.29 (18.42, 22.31)	16.52 (13.95, 19.46)	
Drinking status, (%)				0.0958
Low drinking	86.48 (85.15, 87.71)	86.89 (85.44, 88.22)	84.73 (82.07, 87.06)	
Heavy drinking	13.52 (12.29, 14.85)	13.11 (11.78, 14.56)	15.27 (12.94, 17.93)	
Physical Activity, (%)				0.0168
Insuffciently active	36.85 (35.40, 38.33)	35.84 (34.25, 37.46)	41.17 (37.15, 45.31)	
Active	63.15 (61.67, 64.60)	64.16 (62.54, 65.75)	58.83 (54.69, 62.85)	
Hypertension, (%)				<0.0001
No	71.48 (69.94, 72.98)	73.87 (72.27, 75.41)	61.30 (57.58, 64.88)	
Yes	28.52 (27.02, 30.06)	26.13 (24.59, 27.73)	38.70 (35.12, 42.42)	
Diabetes, (%)				<0.0001
No	93.59 (92.79, 94.30)	94.35 (93.54, 95.06)	90.32 (88.13, 92.14)	
Yes	6.41 (5.70, 7.21)	5.65 (4.94, 6.46)	9.68 (7.86, 11.87)	
Serum Uric Acid (mg/dL)	5.38 (5.34, 5.43)	4.95 (4.92, 4.99)	7.21 (7.14, 7.28)	<0.0001
Triglyceride (mmol/L)	1.24 (1.21, 1.26)	1.16 (1.13, 1.19)	1.55 (1.48, 1.63)	<0.0001
